# Supplementary material for: Bacillus safensis FO-36b and Bacillus pumilus SAFR-032: a whole genome comparison of two spacecraft assembly facility isolates
Source: BMC Microbiol. 2018 Jun 8;18:57. doi: 10.1186/s12866-018-1191-y (PMC5994023; doi:10.1186/s12866-018-1191-y)
Supplement: Supplementary file 2 — Table S2. Presence and absence of the B. safensis FO-36b CRISPR module element protein(s) in the other B. pumilus / B. safensis genomes. (PDF 6 kb) [file 12866_2018_1191_MOESM2_ESM.pdf]

Organism	CRISPR module RAMP protein Cmr1	CRISPR module-associated protein Cas10/Cmr2	CRISPR module-associated protein Cmr3	CRISPR module RAMP protein Cmr4	CRISPR module-associated protein Cmr5	CRISPR module RAMP protein Cmr6
B. safensis **FO-36b^{T}**	**RS87_04350**	**RS87_04355**	**RS87_04360**	**RS87_04365**	**RS87_04370**	**RS87_04375**

List of genomes in which the CRISPR module element(s) is/are absent			List of genomes in which the CRISPR module element(s) is/are present	
B. safensis **strains**	***B. pumilus*** **strains**	***Bacillus*** **sp.**	***B. safensis*** **strains**	***B. pumilus*** **strains**
AFS067952	B4129	WP8	7783	B4107
JPL-MERTA-8-2	B4134		bcp62	B4127
13L_LOBSAL	3-19		bcs96	B4133
Ingolstadt	7P		BRM1	15.1
KCTC12796BP	B6033		BsafCFA06	CB01
MROC	BA06			PDSLzg-1
RP10	BONN			RI06-95
S9	C4			SCAL1
U14-5	CCMA			
U17-1	Fairview			
U41	GM3FR			
VK	GR-8			
RIT	INR7			
	JRS3			
	ku-bf1			
	LK12			
	LK21			
	LK32			
	NCTC10337			
	NJ-M2			
	NJ-V			
	NJ-V2			
	PE09-72			
	S1			
	SF214			
	SH-B9			
	SH-B11			
	TUAT1			
	UBA4042			
	UBA5643			
	W3			
	36R_ATNSAL			
	SAFR-032			
	ATCC7061^{T}			
	GLB197			
	KL4			
	51_5il			
