# Supplementary figures and images for: Bacillus safensis FO-36b and Bacillus pumilus SAFR-032: a whole genome comparison of two spacecraft assembly facility isolates
Source: BMC Microbiol. 2018 Jun 8;18:57. doi: 10.1186/s12866-018-1191-y (PMC5994023; doi:10.1186/s12866-018-1191-y)

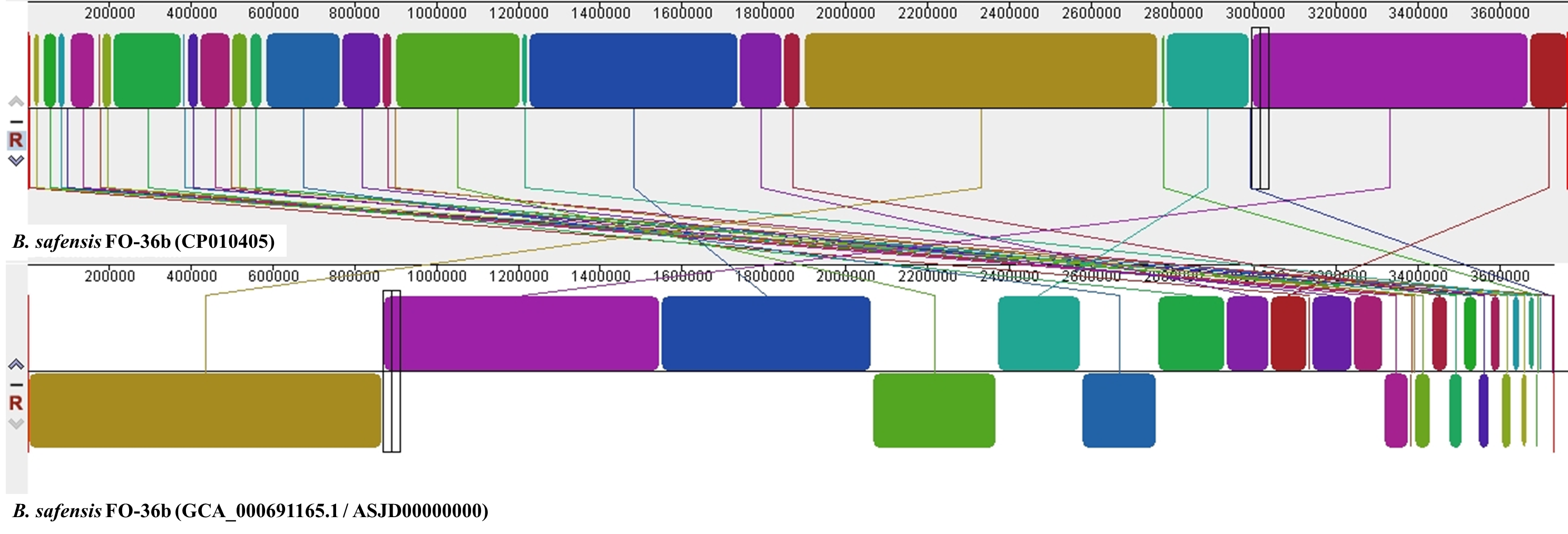

Supplement: Supplementary file 5 — Figure S1. Whole genome alignment of the previously existing B. safensis FO-36b sequence (GCA_000691165.1 / ASJD00000000) with our current updated sequence (CP010405) using Mauve [70]. (TIF 1455 kb) [file 12866_2018_1191_MOESM5_ESM.tif]

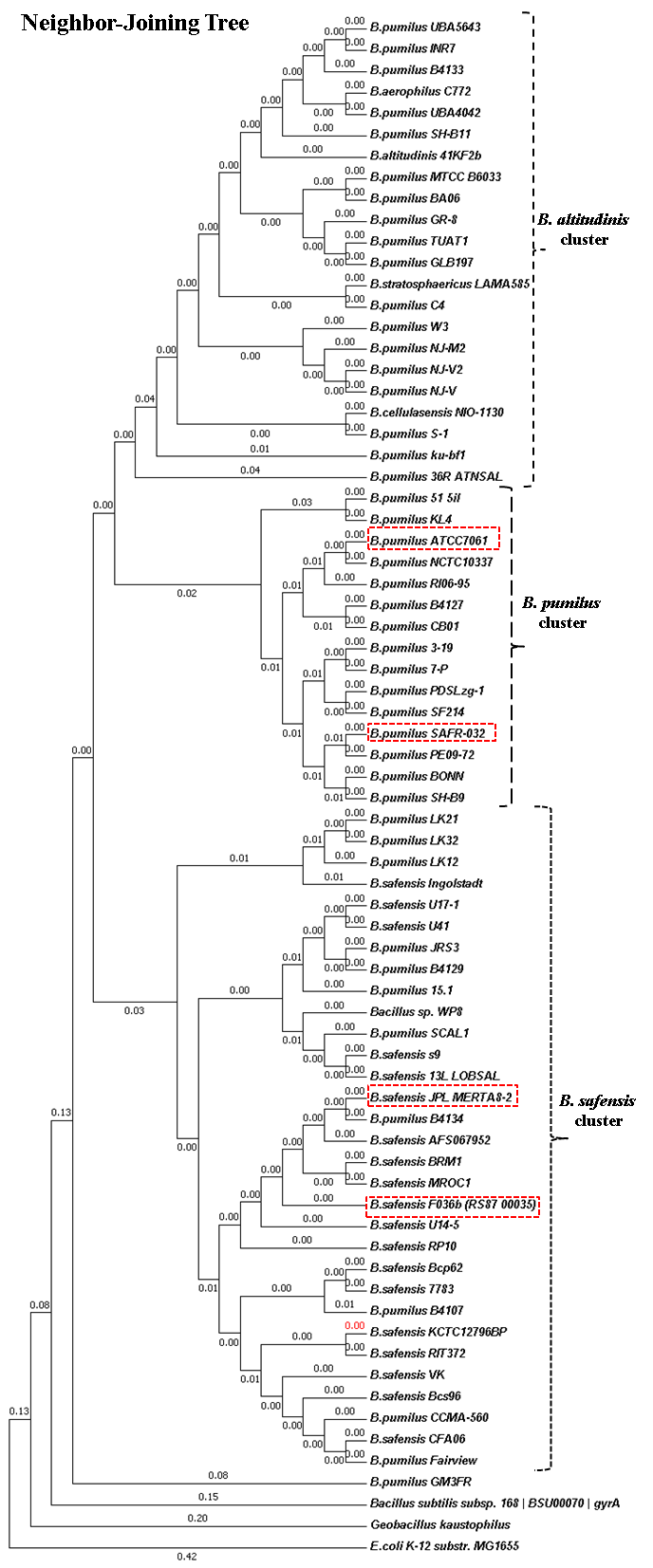

Supplement: Supplementary file 6 — Figure S2. Molecular Phylogenetic analysis by the Neighbor-Joining method. B. safensis FO-36b, B. safensis JPL_MERTA8-2B, B. pumilus SAFR-032, and B. pumilus ATCC7061T are highlighted in red dash-lined rectangles. (TIF 276 kb) [file 12866_2018_1191_MOESM6_ESM.tif]

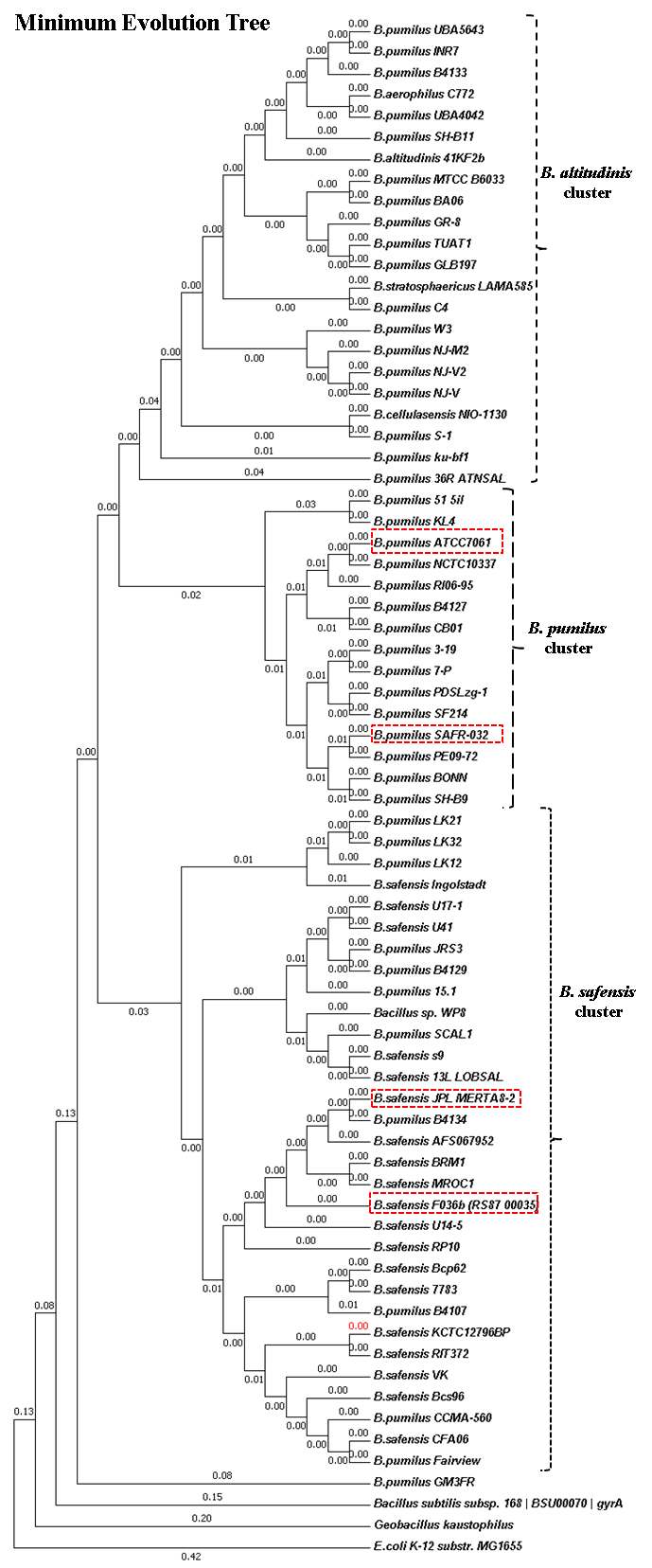

Supplement: Supplementary file 7 — Figure S3. Molecular Phylogenetic analysis using the Minimum Evolution method. B. safensis FO-36b, B. safensis JPL_MERTA8-2B, B. pumilus SAFR-032, and B. pumilus ATCC7061T are highlighted in red dash-lined rectangles. (TIF 275 kb) [file 12866_2018_1191_MOESM7_ESM.tif]
